# Painless Swelling of the Left Jaw in a Two-Year-Old Boy

**DOI:** 10.5334/jbr-btr.1220

**Published:** 2017-02-20

**Authors:** Sofie Woussen, Edwin Van Ovost, Herman Willekens, Marc Lemmerling

**Affiliations:** 1 AZ Sint-Lucas Hospital, Ghent, BE; 2 University Hospital, Ghent, BE

**Keywords:** mandible, chronic osteomyelitis, suppurative osteomyelitis, biopsy

## Abstract

We present a case of a 2-year-old boy with chronic suppurative osteomyelitis of the left jaw. A computed tomography (CT) scan demonstrated a periosteal reaction on the left side of the mandible with cortical destruction of the left mandibular head. The diagnosis could be confirmed histologically. During biopsy of the mandibular lesion, a purulent exudate was withdrawn.

## Case report

**Figure 1:**
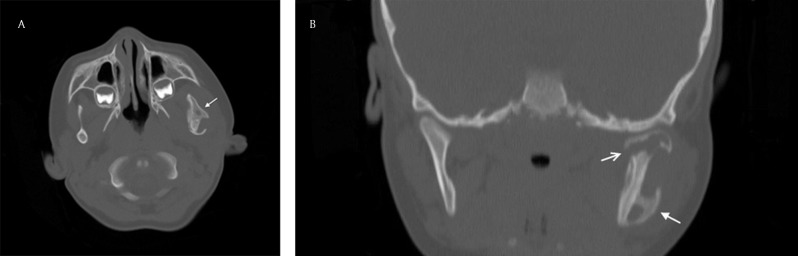
**(A)**; Axial CT image of the mandible in bone window settings showing a sclerotic thickening of the left posterolateral portion of the mandible with parallel periosteal reaction (solid white arrow); **(B)** Coronal reformatted CT image of the mandible in bone window settings showing a sclerotic thickening of the left posterolateral portion of the mandible with parallel periosteal reaction (solid white arrow) and a cortical disruption of the left mandibular head (hollow white arrow).

**Figure 2:**
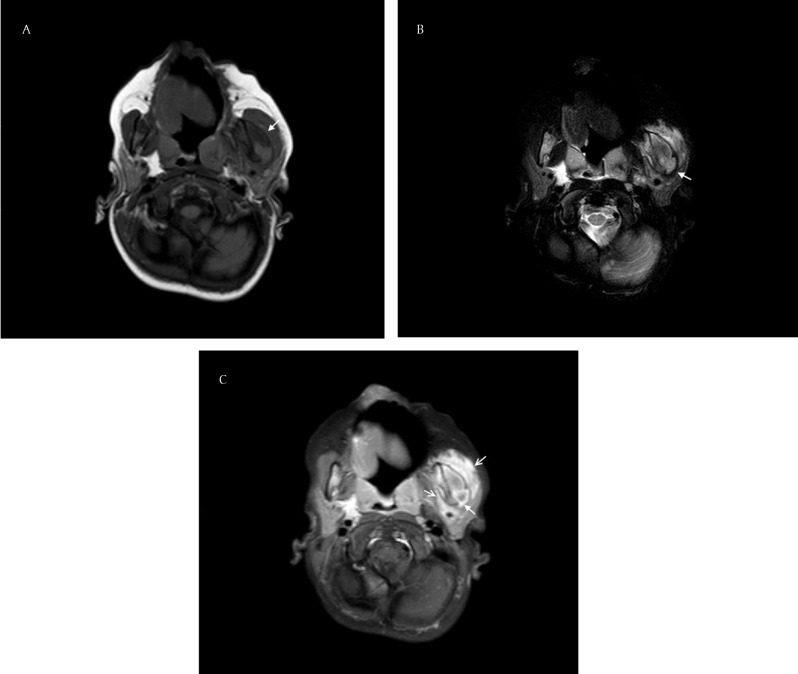
**(A)** T1-weighted axial image of the mandible demonstrating a bone reaction on the left side of the mandible with low signal (solid white arrow); **(B)** Fat-suppressed T2-weighted axial image of the mandible showing a bone reaction on the left side of the mandible with high signal (solid white arrow); **(C)** Gadolinium-enhanced T1-weighted axial image of the mandible, enhancement of Gadolinium is seen in the bone (solid white arrow) and in the adjacent tissues (hollow white arrows).

A two-year-old boy presented with a painless thickening of the left posterolateral portion of the mandible. The swelling had developed progressively over two months. In general, the child was in a good condition. On physical examination, the swelling of the left jaw felt fixed, hard and painless. Intra-oral inspection was unremarkable. Neither tooth decay nor regional lymphadenopathy was present. Laboratory findings showed anemia due to iron deficiency. A computed tomography (CT) scan demonstrated sclerotic thickening of the left mandibular ramus with periosteal new bone formation in a parallel pattern (Fig. 1A and B), and cortical destruction of the left mandibular head (Fig. 1B). No carious teeth were seen. Magnetic resonance imaging (MRI) confirmed a bone reaction on the left side of the mandible that showed low signal on the T1-weighted images (Fig. 2A), high signal on the fat-suppressed T2-weighted images (Fig. 2B) and enhancement after intravenous injection of Gadolinium (Fig. 2C). An inflammatory reaction of the surrounding muscles of mastication was also noted (Fig. 2C). The radiologic findings suggested the diagnosis of chronic osteomyelitis. To confirm the presumed diagnosis and to discriminate malignancy (Ewing sarcoma DD Burkitt lymphoma), a biopsy of the mandibular lesion was performed. During biopsy, a purulent exudate was withdrawn. Histologically bone trabeculae, fibrous intertrabecular stroma and foci of inflammatory cells were identified. Cytologic atypia was not seen. The diagnosis of a chronic suppurative form of osteomyelitis was confirmed. A bacteriologic examination of the specimen was positive for Prevotella oris and Veillonella atypica, members of the oral flora. A two-week treatment with IV amoxicillin/clavulanic acid was initiated followed by a four-week treatment with peroral antibiotics. After six weeks of therapy, the swelling of the left jaw was no longer visible. An MRI showed a partial regression of the lesion.

## Discussion

Our case illustrates an example of the rare suppurative form of a primary chronic osteomyelitis of the left jaw in a two-year-old boy. Osteomyelitis present for at least one month is termed chronic; the mandibular swelling was already present for two months in our case [1]. The most common site for osteomyelitis of the jaws to arise is the posterior body of the mandible [1], as in our case. It should be stressed that the secondary form of chronic osteomyelitis is by far much more common.

CT of the mandible demonstrated some typical radiologic features of osteomyelitis described in the literature: a sclerotic thickening of the bone with parallel periosteal reaction (Fig. 1A and B) and cortical disruption, in this case at the level of the left mandibular head (Fig. 1B) [1]. No bony sequester was present [1]. Practical reasons did not allow us to use a cone beam CT in this case, though it is better to use this modality to ensure a lower radiation dose in children [1]. We also performed an MRI to better show the involvement of the adjacent tissues. The MRI confirmed an extension of the bone inflammation to the left masseter and lateral pterygoid muscles (Fig. 2C). The bone marrow itself showed signs of inflammation.

The laboratory findings in patients with chronic osteomyelitis of the jaw are nonspecific and are mostly limited to an increase of ESR and/or CRP [1]. In our patient, the inflammatory parameters even remained normal.

To obtain a definitive diagnosis and to exclude malignancy, we performed a biopsy of the mandibular lesion. The withdrawal of pus during biopsy already suggested an infectious cause. Histologic examination revealed bone trabeculae, fibrous intertrabecular stroma and foci of inflammatory cells (neutrophil granulocytes). Biopsy specimens were conclusive for oral bacteria that were sensitive for amoxicillin/clavulanic acid. The treatment was effective with regression of the mandibular lesion.
